# Frequency-resolved Monte Carlo

**DOI:** 10.1038/s41598-018-24975-y

**Published:** 2018-05-03

**Authors:** Juan Camilo López Carreño, Elena del Valle, Fabrice P. Laussy

**Affiliations:** 1 Departamento de Física Teórica de la Materia Condensada, Universidad Autónoma de Madrid, 28049 Madrid, Spain; 2 Faculty of Science and Engineering, University of Wolverhampton, Wulfruna St, WV1 1LY United Kingdom; 3 Russian Quantum Center, Novaya 100, 143025 Skolkovo, Moscow Region, Russia

## Abstract

We adapt the Quantum Monte Carlo method to the cascaded formalism of quantum optics, allowing us to simulate the emission of photons of known energy. Statistical processing of the photon clicks thus collected agrees with the theory of frequency-resolved photon correlations, extending the range of applications based on correlations of photons of prescribed energy, in particular those of a photon-counting character. We apply the technique to autocorrelations of photon streams from a two-level system under coherent and incoherent pumping, including the Mollow triplet regime where we demonstrate the direct manifestation of leapfrog processes in producing an increased rate of two-photon emission events.

## Introduction

The Monte Carlo method, of estimation by random sampling, was invented by Ulam as a practical way to estimate the chance of winning a game of solitaire, that a direct combinatorial approach had proved too challenging for the then convalescing scientist who was playing cards on a sick leave^1^. Since this had direct applications for the more “serious” problems tackled at Los Alamos (this was in 1946), such as the computation of neutron diffusion, the activity had to receive a codename, which came after the Monaco ward, famous for its casino. The technique indeed relies on chance by sampling randomly to get hold of a small but representative enough sample to describe a system. This is a surprisingly powerful technique that combines efficiency and accuracy, with applications in virtually all fields of human endeavours^2^.

In quantum physics, the Quantum Monte Carlo technique finds many ramifications in several fields^3^. In the context of interest in this work, that of quantum optics, several methods have been developed in the early 90s (see ref.^4^ for a review). Of these, the quantum jump approach^5–8^ (see Ref.^9^ for an introduction) is particularly appealing as it links the wavefunction collapse to the emission of a photon. Assuming an ideal detector covering the full 4*π* solid angle surrounding the emitter, this allows to perform a computer experiment of photo-detections. From such “clicks” (as we will call a detected photon), one can for instance compute the Glauber correlation functions *g*^(*n*)^ that measure the deviations of intensity correlations at the *n*th order from uncorrelated light, but one can also compute less easily accessible quantities such as exclusive probability densities, e.g., detecting the next photon at a time *τ* after one detection, with no other photon in between (*g*^(2)^ assumes any photon rather than the next one), or distributions of time delays between nearest neighbours, probabilities to detect any given number or even configuration of photons in a time window, or any other type of binning “experiment”. Such Quantum Monte Carlo-generated photons have also been used to support the introduction of the *N*-photon “bundle”^10^, to distinguish the case of *N*-photon sources from strongly-correlated emission at the *N* photon level.

In this text, we apply the quantum-jump Monte Carlo technique to the case of filtered emission, that is to say, as applied to a stream of photons going through an interference (i.e., Lorentzian) filter. Mean values for the correlators can be conveniently obtained with the theory of frequency-resolved photon-correlations^11^. This theory predicts strong correlations in frequency windows that had been neglected by both theorists and experimentalists until recently^12,13^, as they lie far away from the luminescence peaks. Such correlations can clearly be turned into a resource and when technology will be mature to exploit photons as qubits, this aspect will certainly become compelling. As it should be useful to go beyond mean values and get access to time series for a variety of purposes, one could turn to the Quantum Monte Carlo technique applied to color-resolved photons. While the quantum Monte Carlo method has been used to compute the power spectrum as well as time-series of photon emission^14–20^, its combined use for both time *and* energy-resolved photons has, to the best of our knowledge, not been provided before in both a practical and exact form, as the few attempts in this direction^21,22^ have involved a cavity in the weak-coupling limit, which comes at the price of vanishing signal or approximate correlations if coupling of the filter is not weak enough. Monte Carlo methods have been used instead for their computational advantage or to access particular configurations such as the resonance fluorescence spectrum of an effective-three level system in a bright period exclusively (such systems are noted for their intermittent emission). In contrast, our approach allows to extract streams of photons from any frequency windows of a quantum source, using all the signal theoretically available and taking into account self-consistently the effect of its filtering, with an exact treatment of its effect on the correlations. This allows to revisit photon-counting experiments with the added energy degree of freedom, that are already challenging without the frequency constrains.

In this text, we first prove that the technique is exact and, subsequently, we cover two cases for illustration. Namely, we show the effect of filtering a two-level system, and describe how this spoils the antibunching and quantum character of such sources in a practical context, although from a theoretical point of view, the saturated emitter is the brightest single-photon emitter, but of photons with wildly fluctuating frequencies (so not indinstinguishable). We illustrate how the loss of antibunching in time deviates notably from the single-exponential approximation used in the literature^23^. We compare both the cases of coherent and incoherent excitations at low pumping. Then, at high pumping in the coherent case, thus bringing the problem in the Mollow triplet regime, we show how this turns a simple system into a versatile, tunable quantum source, with applications such as quantum spectroscopy^24–26^ or photon sources with tunable statistics^27^. We will also address arguments^28^ that imply that the strongly correlated emission is an artifact of normalization, which we will rebute by explicitly exhibiting these strongly correlated photons thanks to the Monte Carlo simulations. Before that, however, we briefly summarize the theory of frequency-resolved photon correlations and its main conclusions, which are to be found in greater details elsewhere^11,12,22,24,29–33^, and prove that the proposed Monte Carlo method scheme is Mathematically equivalent to this exact theory.

## Theory

Although the process of filtering the light emitted from an optical source has a clear interpretation–the emitted photons are detected in a certain frequency window–its theoretical description used to be far from trivial^34^. With the introduction of the sensor method^11^, this became a straightforward task no more complicated than any old problem of computing quantum correlators, getting rid of all the complicated tasks of normal-ordering and time-integrals in spaces of many dimensions. The technique relies on coupling the system to sensors with strength *ε* and taking the limit of vanishing coupling. In such a limit, it is enough to consider only two levels of the sensors as their populations remain $\ll 1$, without affecting the system’s dynamics. The computation of normalized correlators results in quantities that are *ε* independent to first order and exact in the limit |*ε*| → 0 (taking, in general, $\varepsilon \in {\mathbb{C}}$). These results are also absolute in the sense that they do not depend on detection efficiency or other details of the measurement, but characterize the source’s emission in given frequency windows. Alternatively^35^, such a coupling can also be made through the so-called “cascaded formalism”^36,37^, that describes the dynamics of “detectors”, which are physical objects with a sizable coupling to the source (unlike sensors that have vanishing coupling) but that also do not alter the dynamics of the source, regardless of how strongly they are affected by it. Each method presents some advantages: the sensor method is straightforward to implement while the cascading formalism allows to characterize the detector’s dynamics beyond normalized correlations. An important difference with respect to computational cost is that while the sensor is typically described by a two-level system, the detector must be described by an harmonic oscillator, since it does get populated, whereas the sensor only acts as a probe in the limit of vanishing coupling. As a result, the sensor always provides exact results as a two-level system while the detector must be truncated high enough to provide a close-enough approximation, which depends on the dynamics of the system, and is therefore a tricky question. Furthermore, when considering cross-correlations, instead of *N* two-level systems, one is dealing with *N* harmonic oscillators and the problem becomes numerically forbidding, while the sensors’ Hilbert space scales as 2^*N*^ which is still tractable for *N* much larger than anything that has been considered so far experimentally. For autocorrelations of the *N*th order with the sensor method, one can also use an harmonic oscillator truncated to *N* excitations instead of *N* two-level systems degenerate in frequency, which is also exact and with no need of checking convergenve for higher truncations, as is the case of the cascaded formalism. So in cases where correlations are requested rather than an actual signal, we believe the sensor method to be preferable, as it is both more efficient and more robust. In the present text, however, we specifically require a signal and will therefore turn to the cascaded formalism.

The mathematical equivalence of the two approaches for normalized autocorrelations can be established as follows. On the one hand, the sensor method “plugs” sensors to the dynamics. Formally, calling *σ* the annihilation operator of a source and *ξ* that of a sensor probing it, we can describe their joint dynamics by a Liouvillian equation

1 $${\partial }_{t}\rho =i[\rho ,{H}_{\sigma }+{\omega }_{\xi }\,{\xi }^{\dagger }\xi +\varepsilon {\sigma }^{\dagger }\xi +{\varepsilon }^{\ast }\sigma {\xi }^{\dagger }]+\frac{{\gamma }_{\sigma }}{2}{ {\mathcal L} }_{\sigma }\rho +\frac{{\rm{\Gamma }}}{2}{ {\mathcal L} }_{\xi }\rho \,,$$

where *H*_*σ*_ is the Hamiltonian that describes the internal dynamics of the source, *γ*_*σ*_ is the decay rate of the source, Γ is the decay rate of the sensor and ${ {\mathcal L} }_{c}\rho \equiv 2c\rho {c}^{\dagger }-{c}^{\dagger }c\rho -\rho {c}^{\dagger }c$. The dynamics of an arbitrary operator ${\xi }^{\dagger \mu }{\xi }^{\nu }{\sigma }^{\dagger m}{\sigma }^{n}$ under the action of this Liouvillian is described with the notations of ref.^11^, by the equation (we set *ħ* = 1 along the paper)

2 $$\begin{array}{ccc}{{\rm{\partial }}}_{t}\overrightarrow{\omega }[\mu ,\nu ] & = & \{M+[(\mu -\nu )i{\omega }_{\xi }-(\mu +\nu )\frac{{\rm{\Gamma }}}{2}]{\bf{1}}\}\overrightarrow{\omega }[\mu ,\nu ]\\  &  & +\,i\varepsilon \mu {T}_{+}\overrightarrow{\omega }[\mu -1,\nu ]-i{\varepsilon }^{\ast }\nu {T}_{-}\overrightarrow{\omega }[\mu ,\nu -1]+O({\varepsilon }^{2}),\end{array}$$

where **1** is the unit matrix, *T*_±_ are normal-ordering superoperators for the *σ* operators and $\overrightarrow{\omega }$[*μ*, *ν*] is a vector of correlators for the *μ*th and *ν*th powers of the sensor operators *ξ*, ${\xi }^{\dagger }$ and spanning in normal order all powers of the *σ*, ${\sigma }^{\dagger }$ operators, i.e., $\overrightarrow{\omega }[\mu ,\nu ]\equiv {(\langle {\xi }^{\dagger \mu }{\xi }^{\nu }\rangle ,\langle {\xi }^{\dagger \mu }{\xi }^{\nu }\sigma \rangle ,\langle {\xi }^{\dagger \mu }{\xi }^{\nu }{\sigma }^{\dagger }\rangle ,\cdots ,\langle {\xi }^{\dagger \mu }{\xi }^{\nu }{\sigma }^{\dagger m}{\sigma }^{n}\rangle ,\cdots )}^{T}$. The *O*(*ε*^2^) notation means that all other terms are of higher order in *ε*. The matrix *M* provides the dynamics for the source, ∂_*t*_$\overrightarrow{\omega }$[0, 0] = *M*$\overrightarrow{\omega }$[0, 0] + *O*(*ε*^2^), and is independent of the sensor at the lowest order in *ε*. At this stage, we do not assume any property of *σ* or *ξ*, which could be bosonic (in which case *μ*, *ν*, *m* and *n* are unbounded) or fermionic (in which case *μ*, *ν*, *m* and *n* are 0 or 1). Equation (2) can be integrated, which yields

3 $$\overrightarrow{\omega }[\mu ,\nu ]=i|\varepsilon |{\{M+[(\mu -\nu )i{\omega }_{\xi }-(\mu +\nu )\frac{{\rm{\Gamma }}}{2}]{\bf{1}}\}}^{-1}(-{e}^{i\theta }\mu {T}_{+}\overrightarrow{\omega }[\mu -1,\nu ]+{e}^{-i\theta }\nu {T}_{-}\overrightarrow{\omega }[\mu ,\nu -1])+O({\varepsilon }^{2})\,,$$

where *ε* = |*ε*|*e*^*iθ*^. This in turn can be solved recursively, down to $\overrightarrow{\omega }$[0, 0] where the equation self-truncates. Each element $\overrightarrow{\omega }$[*μ*, *ν*] is found to be, by inspection of Eq. (3), of the order |*ε*|^*μ*+*ν*^, to the smallest order in |*ε*| (leading when |*ε*| → 0). Note that only the absolute value of the coupling can be extracted as a common factor in Eq. (3). This results in (*μ* + 1)(*ν* + 1) − 1 nested equations and unknowns in order to compute a given element $\overrightarrow{\omega }$[*μ*, *ν*]. More details on this derivation can be found in the Supplementary Material of Ref.^11^. The important point is that normalised *n*th order correlators, ${g}_{{\rm{\Gamma }}}^{(n)}$, are ratios of the first component of $\overrightarrow{\omega }$[*n*; *n*] (that is, $\langle {\xi }^{\dagger n}{\xi }^{n}\rangle$) divided by the *n*-th power of the first component of $\overrightarrow{\omega }$[1; 1] (that is, $\langle {\xi }^{\dagger }\xi \rangle$), itself of order |*ε*|^2^, so that in such a ratio *ε* is cancelled to leading order. Although higher-order terms would spoil this cancellation, they become negligible as the sensor coupling is made smaller. Therefore, in the limit |*ε*| → 0, the result becomes exact.

On the other hand, the cascaded formalism, which aims at exciting a target without affecting the source, provides a similar type of cancellation, although not restricted to vanishing coupling. From a causality point of view, it is clear that such a source/detector scenario where only one affects the other can be realized. The source that emitted a photon towards a detector may not even exist anymore by the time the detector is excited. This is achieved formally through interferences that cancel the back-action from the detector to the source. The master equation describing this asymmetric coupling reads

4 $${\partial }_{t}\rho =i[\rho ,{H}_{\sigma }+{\omega }_{\xi }\,{\xi }^{\dagger }\xi ]+\frac{{\gamma }_{\sigma }}{2}{ {\mathcal L} }_{\sigma }\rho +\frac{{\rm{\Gamma }}}{2}{ {\mathcal L} }_{\xi }\rho +\sqrt{\alpha {\gamma }_{\sigma }{\rm{\Gamma }}}\{[\sigma \rho ,{\xi }^{\dagger }]+[\xi ,\rho {\sigma }^{\dagger }\mathrm{]\}.}$$

The last three-terms of Eq. (4) can be re-written in the Lindblad form as

5 $$\begin{array}{c}\frac{{\gamma }_{\sigma }}{2}{ {\mathcal L} }_{\sigma }\rho +\frac{{\rm{\Gamma }}}{2}{ {\mathcal L} }_{\xi }\rho +\sqrt{\alpha {\gamma }_{\sigma }{\rm{\Gamma }}}\{[\sigma \rho ,{\xi }^{\dagger }]+[\xi ,\rho {\sigma }^{\dagger }]\}\\ \,=\,\frac{1}{2}{ {\mathcal L} }_{\hat{o}}\rho +\frac{{\chi }_{1}{\gamma }_{\sigma }}{2}{ {\mathcal L} }_{\sigma }\rho +\frac{{\chi }_{2}{\rm{\Gamma }}}{2}{ {\mathcal L} }_{\xi }\rho +\frac{\sqrt{\alpha {\gamma }_{\sigma }{\rm{\Gamma }}}}{2}[\rho ,{\xi }^{\dagger }\sigma -{\sigma }^{\dagger }\xi ]\,,\end{array}$$

where $\hat{o}=\sqrt{\mathrm{(1}-{\chi }_{1}){\gamma }_{\sigma }}\sigma +\sqrt{\mathrm{(1}-{\chi }_{2}){\rm{\Gamma }}}\xi$ is the joined decay operator of the whole system, source and detector, and the interpretation of the factor *χ*_*k*_ becomes that of factors that quantify the amount of signal that each part, source and detector, generates on its own and that the joined system generates as a whole. The detector, which must have a finite lifetime to couple to the source, thus also has an intrinsic frequency window with effect of filtering the emission it detects, whence the connection to the sensors formalism. The factor *α* = (1 − *χ*_1_)(1 − *χ*_2_), for 0 ≤ *χ*_1_, *χ*_2_ ≤ 1, takes into account that the source can have several decay channels. This is required for instance when only fluorescence is wanted without contamination from another source, e.g., a laser exciting it (experimentally this is typically achieved by detecting at right angle from the exciting beam).

Our proof proceeds by showing that ${\xi }^{\dagger \mu }{\xi }^{\nu }{\sigma }^{\dagger m}{\sigma }^{n}$ has the same equation as in the sensor formalism, by computing explicitly the equation for ∂_*t*_$\overrightarrow{\omega }$[*μ*, *ν*] in the cascaded formalism, Eq. (5). This reads, to all orders in the coupling in this case:

6 $$\begin{array}{ccc}{{\rm{\partial }}}_{t}\overrightarrow{\omega }[\mu ,\nu ] & = & \{M+[(\mu -\nu )i{\omega }_{\xi }-(\mu +\nu )\frac{{\rm{\Gamma }}}{2}]{\bf{1}}\}\overrightarrow{\omega }[\mu ,\nu ]\\  &  & -\,\sqrt{\alpha {\gamma }_{\sigma }{\rm{\Gamma }}}\{\mu {T}_{+}\overrightarrow{\omega }[\mu -1,\nu ]+\nu {T}_{-}\overrightarrow{\omega }[\mu ,\nu -1]\}.\end{array}$$

Remarkably, this equation has the same form as Eq. (2) with $\varepsilon \to i\sqrt{\alpha {\gamma }_{\sigma }{\rm{\Gamma }}}$. Even though *ε* is complex and a vanishing quantity in Eq. (2), with higher order corrections, and $\sqrt{\alpha {\gamma }_{\sigma }{\rm{\Gamma }}}$ is real and finite in Eq. (6), both methods provide exactly the same normalised correlators, as these coupling parameters enter in both the numerators and denominators with the same power and cancel out. The result becomes exact for vanishing coupling in the case of sensors and is exact in all cases with the cascaded formalism, regardless of their normalisation. Note as well that *θ*, the phase of the coupling *ε*, has an effect on the dynamics only if the Lindblad equation features products of different operators in its dissipative terms, which is the case for the cascaded formalism with ${ {\mathcal L} }_{\hat{o}}$ that brings cross terms of *σ* and *ξ*. The sensor formalism, however, has no such joint decay emission and the phase of *ε* does not play any role, so that *ε* could have been set real. This achieves to prove the mathematical equivalence of the sensor method with the cascaded formalism for the computation of normalized correlators.

**Figure 1:**
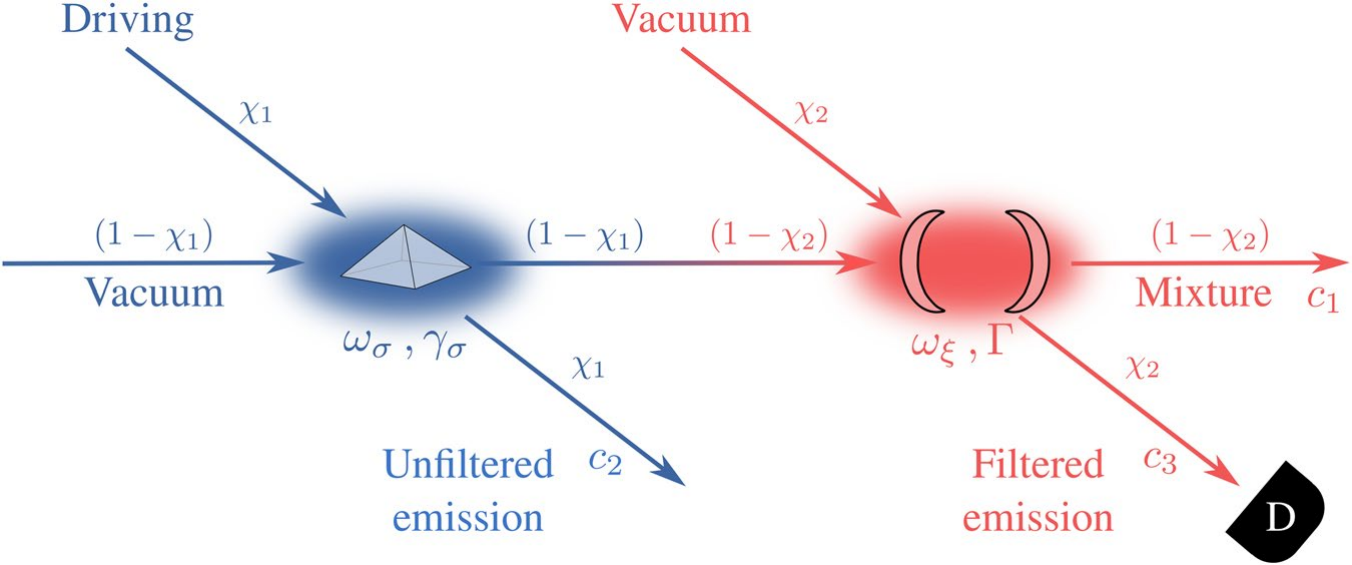
Scheme of the setup to measure the frequency-resolved correlations of the light emitted by a source, whose energy is centered at *ω*_*σ*_ and has a decay rate *γ*_*σ*_. While a fraction of the emitted light goes unfiltered to the open space, to which we refer to as the “unfiltered emission” and which is described by the quantum jumps of the operator *c*_2_ in Eq. (7), the remaining fraction is used to weakly drive the sensor, which has frequency *ω*_*ξ*_ and decay rate Γ, which is also the bandwidth of the sensor. The emission from the sensor can also be separated into two streams, depending on whether the emission from the sensor is mixed or not with scattered light from the source (e.g., the emission of the sensor and the scattered light might follow different spatial paths). The case without the scattered light corresponds to the “filtered emission” which can then go to a detector D or a Hanbury Brown-Twiss setup. It is associated to the operator *c*_3_ of Eq. (7), while the mixture of light is described through the operator *c*_1_ of Eq. (7).

Since the sensor formalism has been shown^11^ to be equivalent to normalized photon correlations according to photo-detection theory^34^, the above equivalence of the sensor and cascaded formalisms shows that applying the quantum Monte Carlo method to the detector^38^ realises a sampling of the emission in the corresponding frequency windows, from which one can reconstruct the frequency-resolved photon correlations. That is to say, this allows us to simulate the photon emission with both time and energy information, which is what we are going to illustrate in the following. Note that with both Eqs (2) and (6), any given correlator $\langle {\xi }^{\dagger \mu }{\xi }^{\nu }\rangle$ can be computed exactly (by recurrence) in terms of lower order ones $\langle {\xi }^{\dagger \mu ^{\prime} }{\xi }^{\nu ^{\prime} }\rangle$ only, with *μ*′ ≤ *μ* and *ν*′ ≤ *ν*. This means that both methods can be applied using *N* two-level systems as detectors at different frequencies, in order to compute cross correlations, or with a single harmonic oscillator truncated at *N* excitations, to compute the *N*th order monocromatic autocorrelation function ${g}_{{\rm{\Gamma }}}^{(N)}$. This is however not sufficient with the cascaded formalism for computing the density matrix (full state) of the detectors or for doing Monte Carlo simulations of the emission. In such cases, one must model the *N* detectors as harmonic oscillators with a high enough truncation to provide converged results. The simulation is conveniently implemented through the quantum-jump approach. The dynamics of the system is thus described by a wavefunction $|\psi (t)\rangle$ that occasionally undergoes a process of “collapsing”, attributed to the emission of a photon, that one records in the simulation as a detector would register a click in an experiment. The collapse is decided in each infinitesimal time interval *δt* → 0, where the evolution of the wavefunction is governed by two elements: a non-Hermitian Hamiltonian and random quantum jumps. In a system described by the master equation ${\partial }_{t}\rho =i[\rho ,H]+\mathrm{(1/2)}{\sum }_{k}\,{ {\mathcal L} }_{{c}_{k}}\rho$, the non-Hermitian Hamiltonian is constructed as $\tilde{H}=H-(i\mathrm{/2)}{\sum }_{k}{c}_{k}^{\dagger }{c}_{k}$, and the evolution of the wavefunction is given either by

$$|\psi (t+\delta t)\rangle =\frac{{c}_{k}|\psi (t)\rangle }{\langle \psi (t)|{c}_{k}^{\dagger }{c}_{k}|\psi (t)\rangle }\,{\rm{o}}{\rm{r}}\,|\psi (t+\delta t)\rangle =\frac{\exp (i\mathop{H}\limits^{ \sim }\delta t)|\psi (t)\rangle }{\langle \psi (t)|\exp (i{\mathop{H}\limits^{ \sim }}^{\dagger }\delta t)\exp (i\mathop{H}\limits^{ \sim }\delta t)|\psi (t)\rangle }\,,$$

depending on whether the system undertook or not a quantum jump, respectively. The probability that this happened in the interval ~*δt* due to the operator ~*c*_*k*_ is proportional to the mean value of this operator, namely ${p}_{k}=\langle \psi |{c}_{k}^{\dagger }{c}_{k}|\psi \rangle \delta t$. For the system described by the master equation (4), the collapse operators are (cf. Fig. 1)

7 $${c}_{1}=\sqrt{(1-{\chi }_{1}){\gamma }_{\sigma }}\,\sigma +\sqrt{(1-{\chi }_{2}){\rm{\Gamma }}}\,\xi \,,\,{c}_{2}=\sqrt{{\chi }_{1}{\gamma }_{\sigma }}\,\sigma \,{\rm{a}}{\rm{n}}{\rm{d}}\,{c}_{3}=\sqrt{{\chi }_{2}{\rm{\Gamma }}}\xi ,$$

whereas the non-Hermitian Hamiltonian is given by

8a $$\begin{array}{rcl}\tilde{H} & = & {H}_{\sigma }+{H}_{\xi }-\frac{i}{2}\sqrt{\alpha {\gamma }_{\sigma }{\rm{\Gamma }}}({\xi }^{\dagger }\sigma -{\sigma }^{\dagger }\xi )-\frac{i}{2}({c}_{1}^{\dagger }{c}_{1}+{c}_{2}^{\dagger }{c}_{2}+{c}_{3}^{\dagger }{c}_{3}),\end{array}$$

8b $$\,=\,{H}_{\sigma }+{H}_{\xi }-i\sqrt{\alpha {\gamma }_{\sigma }{\rm{\Gamma }}}{\xi }^{\dagger }\sigma -\frac{i}{2}({\gamma }_{\sigma }{\sigma }^{\dagger }\sigma +{\rm{\Gamma }}{\xi }^{\dagger }\xi \mathrm{).}$$

The Monte Carlo approach within the cascaded formalism context has already been considered^37^ but without connecting it to frequency-resolved correlations. Now that we have established this correspondence mathematically, we illustrate in the following how the clicks collected through Eqs. (4,5), in the frequency window determined by the detector, match indeed with the correlations predicted by the theory of frequency-resolved photon correlations^11^. Here, we apply this technique to the driven two-level system, under both coherent and incoherent emission, at low and large pumping. While some of the underlying physics has been published elsewhere, this will allow us to revisit it from another angle and provide additional results. We will consider both the cases of autocorrelations and cross-correlations. Although the same principles could be extended to even more than two detectors, we postpone such discussions and their further applications to future works.

### The two-level system

**Figure 2:**
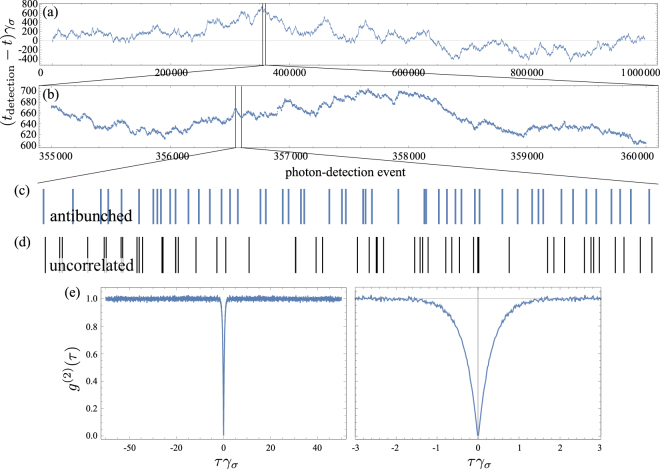
Monte Carlo method on a two-level system. (**a**) Times of emission for 1 000 000 recorded photons as compared to their mean emission rate, exhibiting a classical random walk. (**b**) Zoom of (a) in the highlighted window. (**c**) Zoom of (b) in the highlighted window, with detected photons now displayed in absolute time rather than relatively to their mean emission time. Locally, one can observe a structure in their statistical distribution, with a tendency of ordering and mutual repulsion. This becomes obvious when comparing with uncorrelated photons with the same emission rate, shown in (**d**). The latter exhibit Poisson bursts. (**e**) Intensity correlations *g*^(2)^(*τ*) computed from the one million points, in two timescales, featuring a clear antibunching. The inverse lifetime of the two-level system sets the time unit.

The simplest system in quantum physics is the two-level system. We find it inspiring that it is still a Research problem, constantly posing new questions. Manifestly, in a quantum universe, the two-level system is as complicated as anything else. We first discuss a very basic problem, namely, the effect of filtering on the photon emission from the Monte Carlo point of view. Starting with incoherent pumping, at rate *P*_*σ*_, with Hamiltonian

9 $${H}_{\sigma }={\omega }_{\sigma }{\sigma }^{\dagger }\sigma \,,$$

for a two-level system with free energy *ω*_*σ*_, and Liouvillian

10 $${\partial }_{t}\rho =i[\rho ,{H}_{\sigma }]+({\gamma }_{\sigma }\mathrm{/2)}{ {\mathcal L} }_{\sigma }\rho +({P}_{\sigma }\mathrm{/2)}{ {\mathcal L} }_{{\sigma }^{\dagger }}\rho \,,$$

with *γ*_*σ*_ the inverse lifetime, one finds a simple enough dynamics of Glauber’s second order correlator:

11 $${g}^{\mathrm{(2)}}(\tau )=1-\exp [\,-\,({\gamma }_{\sigma }+{P}_{\sigma })\tau ]\,,$$

with, in particular, *g*^(2)^(0) = 0, that is, perfect antibunching. A (conventional) Monte Carlo simulation using the technique explained above, is shown in Fig. 2. The upper panel shows the fluctuations in the detection times of a million photons from such a source. As such, this realizes a random walk, similar to a random (Poissonian) process, and at large timescales there is nothing noticeable. On the short timescale, however, one can observe clear correlations of antibunching, as shown in the series of clicks indicated by blue ticks in Fig. 2(c). Namely, photons tend to repel each other and appear more orderly than if they would be uncorrelated, as is the case of the second series of photon detections, shown for comparison with black ticks in Fig. 2(d). The uncorrelated series exhibits the counter-intuitive “Poisson clumping” or “Poisson burst” effect^39^, made famous by von Bortkiewicz’s horse kicking casualties in the Prussian army and still of recurrent appearance in the medias as intuition repels the notion that a burst of accidents in, say, a hospital, is a natural random process rather than negligence. The strongly-correlated character of the two-level system emission becomes clear and compelling when computing intensity correlations *g*^(2)^(*τ*) from the clicks, defined as the density of probability of finding two photons with a time difference *τ*. Specifically, from the times of detection *t*_*i*_, we compute *t*_*i*_ − *t*_*j*_ for all 1 ≤ *i* ≤ *N* with *N* the total number of detected photons (here *N* = 10^6^) and compare the density of time differences to that from uncorrelated clicks with the same intensity. Note that in a typical experiment, a first photon starts a timer and a second stops it, and a distribution of the time difference between successive photons is used as a good approximation. In our case, we compute the exact correlations by collecting all the time differences within the correlation window of interest. This is shown for |*τ*| ≤ 50/*γ*_*σ*_ in Fig. 2(e), left. One sees an overall plateau, indicating that photons have the same distribution for long-time separations as if they were emitted by a Poisson process (randomly). But one also observes a clear dip at *τ* ≈ 0, indicating that at such close distances, photons behave very differently than uncorrelated ones, namely, the occurrence of small time delays is strongly suppressed. This is better resolved in Fig. 2(e), right. Such a behaviour defines antibunching, *g*^(2)^(0) < *g*^(2)^(*τ*), with coincidences, i.e., simultaneous detection of two photons, less likely to occur than other closely spaced detections, with perfect suppression of coincidences when *g*^(2)^(0) = 0. Since these correlations wash out at long times, one has ${\mathrm{lim}}_{\tau \to \infty }\,{g}^{\mathrm{(2)}}(\tau )=1$. The time it takes to reach this plateau is the second-order coherence time. We do not need to overlap these results of the Monte Carlo signal with the theory curve, Eq. (11), since, with one million points, it is exact to within the plot accuracy. Beside the statistical noise, that starts to be apparent for $\tau  > \mathrm{1/(2}{\gamma }_{\sigma })$, the Monte Carlo data provides a smooth curve in the window of strong correlations. In our simulation, the Δ*t* was 0.01/*γ*_*σ*_ and the binning size was taken twice as large, corresponding to the two closely-spaced vertical lines on the right panel of Fig. 2(e), bounding *g*^(2)^(0) from below due to this small uncertainty. With a binning size equal to the Monte Carlo timestep, one recovers the perfect antibunching at the origin, although on two grid points, so also producing a small error (the result would be perfect only in the limit of vanishing timesteps).

These results provide the background for our approach in the filtered signal. The general question is, what happens to the emitted photons if a filter is interposed on their way to the detector? This does not simply subtract a fraction, it also redistributes those that make it through, to provide them with possibly very different statistical properties, as we now discuss in more details.

## Emission of a filtered two-level system

### Incoherent excitation

**Figure 3:**
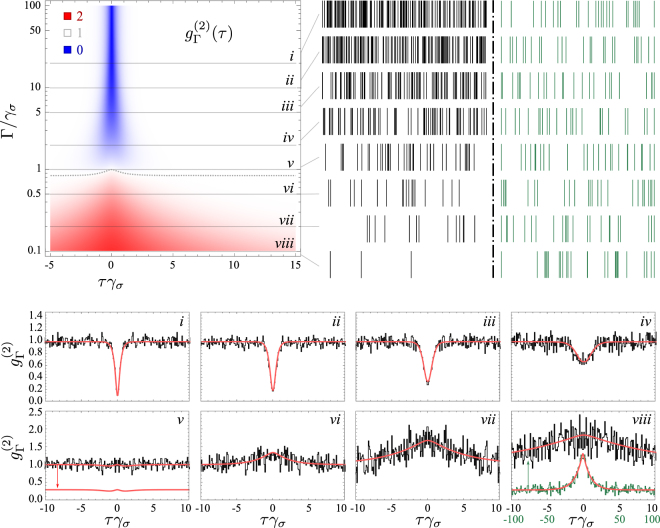
Frequency-resolved emission from an incoherently driven two-level system. The number of events (clicks) recorded are close to 10 000 (namely 9976, 9916, 9974, 9927, 9967, 9955, 9860) for the cases *i*–*vii* respectively, and 25 000 for case *viii* to get enough signal for the small timescale comparison to the other filters. The density-plot is the theoretical ${g}_{{\rm{\Gamma }}}^{\mathrm{(2)}}(\tau )$ with the color code indicated (blue for antibunching, red for bunching and white for uncorrelated). Filtering leads to thermalization. The transition is slightly more complex than merely loss of antibunching. The dotted line shows the isoline ${g}_{{\rm{\Gamma }}}^{\mathrm{(2)}}(\tau )=1$. Monte Carlo simulations have been done for the eight cuts shown. Samples of clicks are shown in the same time window (black, left) or with rescaling to have the same intensity (green, right). There is a neat transition visible to the naked eye between the two types of photon statistics. Autocorrelation computed from the clicks are shown in the eight panels at the bottom, together with the theory prediction. In panel *v*, the theory curve is also shown displayed to reveal its fine structure departing from ${g}_{{\rm{\Gamma }}}^{\mathrm{(2)}}(\tau )=1$. In panel *viii*, also the case of longer times is shown since thermalization goes together with slowing down of the dynamics. For the density plot 1/*γ*_*σ*_ sets the unit and *P*_*σ*_ = 2*γ*_*σ*_. The clicks correspond to the emission events of the operator *c*_3_ in Eq. (7).

The effect of a Lorentzian filter on the statistics of emission of an incoherently excited two-level system is shown in Fig. 3. The theory predicts thermalization and loss of antibunching with narrowing filtering. The exact way how this happens is discussed elsewhere^23^, and the theoretical result is shown on the density plot in Fig. 3 along with eight Monte-Carlo simulations of roughly 10 000 clicks each (25 000 for the narrowest filter in case *viii*). Extracts of the recorded clicks are shown, comparing them, 1) in the same time window (black ticks), with effect of having much less clicks for narrower filters, and also 2) when rescaling the unit of time so that the intensities are the same (green ticks). In the latter case, one can compare the statistical distributions, and observe the transition from antibunched clicks (*i*) to thermal ones (*viii*) passing by auxiliary distributions. In the former case, one observes the characteristic antibunching, equally-spaced like distribution of a two-level emitter. In the latter case, one finds the wildly fluctuating thermal (or chaotic) light, with pronounced bunching in the form of long gaps of no emission followed by gusts of emission. This can be differentiated even with the naked eye from the Poisson distribution, whose tendency for “clumping” does not get as dramatic as the thermal case. One can follow the transition neatly from these various sets of clicks, passing by the case of almost uncorrelated light. Since the isoline ${g}_{{\rm{\Gamma }}}^{\mathrm{(2)}}(\tau )=1$ is not straight (it is shown as a dotted line in the density plot of Fig. 3), the passage from antibunching to bunching does not transit through exactly uncorrelated (or coherent light), although the deviation is too small to be appreciated on a small sample. To observe such fine variations, one needs to acquire a large statistical ensemble and condense the correlations in a single object, such as ${g}_{{\rm{\Gamma }}}^{\mathrm{(2)}}$, as is shown in the eight panels at the bottom of Fig. 3. The case *v* of close-to-uncorrelated light is also shown separately from the Monte Carlo data to reveal its fine structure. The other cases have a simpler shape of a dip that turns into a hump. The correlation time also changes dramatically, as is observable both from the density plot and the Monte Carlo histograms. As the emission thermalizes, its fluctuations occur on longer timescales. This is the reason for the increased noise in panels *vi*–*viii*. There, one should increase the binning and consider larger time windows, as shown in green for case *viii* that assumes a binning of Δ*tγ*_*σ*_ = 1 instead of 0.1 for the other cases, and plot the correlations in a time window |*tγ*_*σ*_| ≤ 100 instead of 10, as indicated on the respective axes, recovering the excellent agreement with theory displayed by the antibunched cases.

Now in possession of the statistical data, and with the insurance of its accuracy given its agreement with the theory, it is possible to undertake various types of analysis that would not be so straightforward theoretically, as has been described in the introduction. We will not go in this direction now and leave for future works and/or other colleagues (the statistical data is available on a public repository^40^). Instead, we now turn to the case of coherent excitation, that also presents features of interest.

### Coherent excitation

**Figure 4:**
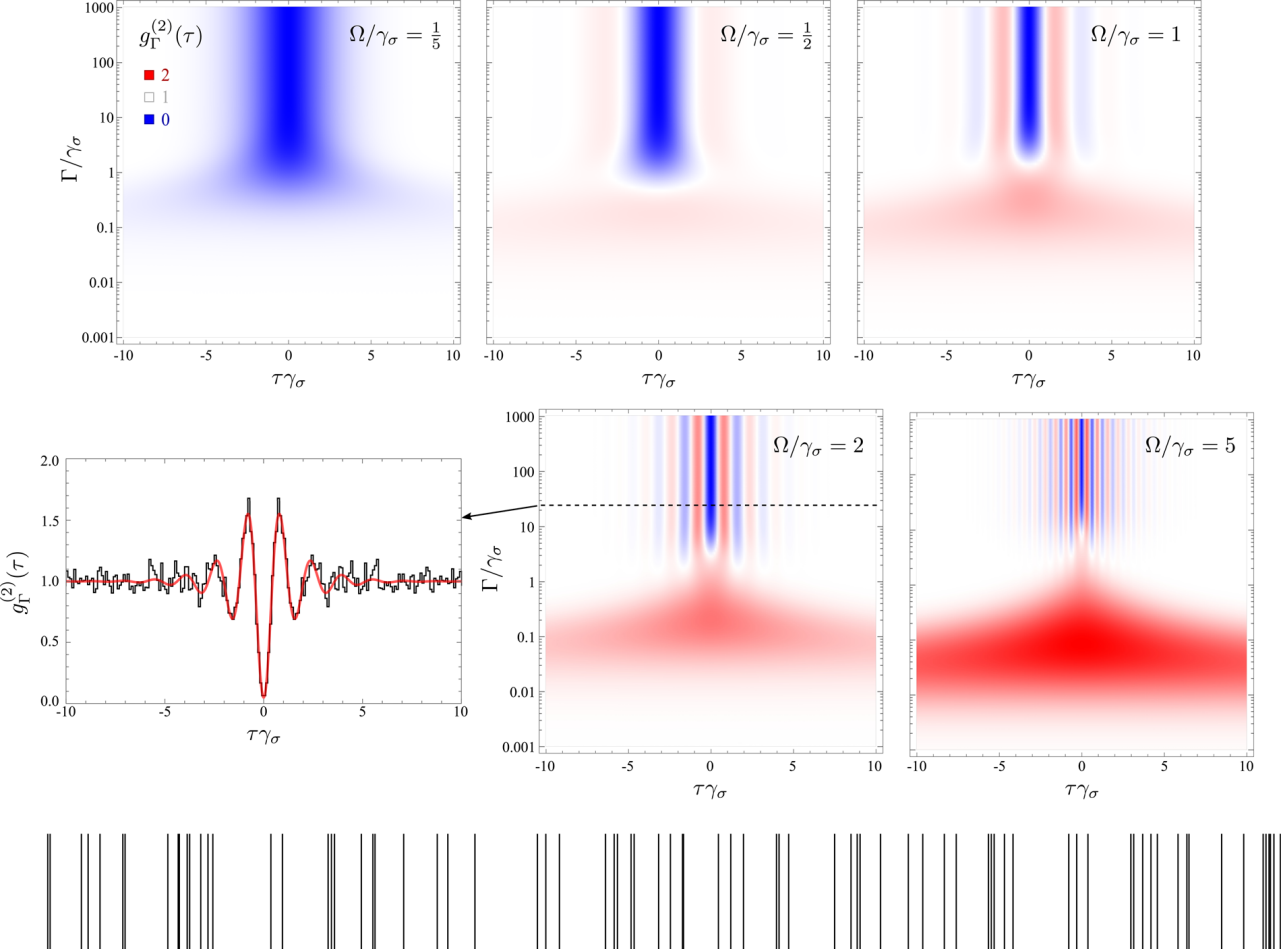
Frequency and time-resolved ${g}_{{\rm{\Gamma }}}^{\mathrm{(2)}}(\tau )$ of a two-level system coherently driven, in its transition from the Heitler to the Mollow regime (from left to right). At low pumping, one does not observe thermalization (bunching) with narrowing filters. Higher pumping brings both bunching, similar to the case of incoherent pumping, and oscillations. The bunching is observed only for moderately narrow filtering as extremely narrow filtering goes back to filtering exclusively the Rayleigh peak, with a resurgence of the Heitler effect and uncorrelated (or coherent) emission. Wide filtering overlapping the three peaks captures the Rabi oscillations. A Monte Carlo simulation of the case highlighted with the dashed line is shown through a small sample of clicks (bottom) and the autocorrelation function, compared to the theory prediction. There is a clear structure in the photon clicks, that is unlike any of the cases shown previously. The clicks correspond to the emission events of the operator *c*_3_ in Eq. (7).

The case of coherent excitation is obtained by complementing the Hamiltonian in Eq. (9) with the driving of the two-level system, ${\rm{\Omega }}({\sigma }^{\dagger }+\sigma )$, and dropping the last term in the master equation (10). The case of filtered coherently driven 2LS is shown in Fig. 4. Here as well, there is thermalization, although this occurs in the case of strong-driving^41^, it is interesting to consider the effect of filtering and approach it from the Monte Carlo perspective. Taking one slice featuring these oscillations, we collect 10^5^ clicks, a small portion of which is shown as ticks at the bottom of Fig. 4. Computing the autocorrelations, we find indeed strong oscillations from a very good antibunching with steep bunching elbows, in agreement with the theory. This produces even more pronounced correlations in the photon-detection events, where the spacing appears more regular and between clumps of photons. As far as continuous streams are concerned, this suggests that such strongly-oscillating *g*^(2)^ do in fact provide more ordered time series than the conventional antibunching of the type of Eq. (11). Such questions are however beyond the scope of the present text. We conclude this Section with further comments on the Heitler effect (coherence of the Rayleigh peak), that is broken at high pumping, but is eventually restored with narrow-enough filtering. First, regarding the emergence of a thermalization similar to that of incoherent driving, cf. Fig. 3, this is obtained when one enters the Mollow triplet regime^42^. In this case, the luminescence has split into a triplet lineshape and, when filtering at resonance (as is the case here), one filters the central peak alone, which is known to correspond to the spontaneous emission of a photon that leaves the state of the dressed two-level system unchanged^43^. As such, the spontaneously emitted photons react to filtering in a similar way than the incoherently pumped two-level system, hence the observed bunching for narrowing filters linewidths. The similarity is only partial, however, as instead of thermalization, with ${g}_{{\rm{\Gamma }}}^{\mathrm{(2)}}\mathrm{(0)}=2$, the transition is to a super-chaotic state, with ${g}_{{\rm{\Gamma }}}^{\mathrm{(2)}}\mathrm{(0)}=3$ in the limit of infinite pumping^12^ (for the parameters considered here, we find ${max}_{{\rm{\Gamma }}}\,{g}_{{\rm{\Gamma }}}^{(2)}(0)\approx 2.2$). More strikingly, when filtering well within the central peak, one then isolates the Rayleigh (*δ*) peak again and reverts to the low-pumping case, with the statistics becoming uncorrelated, as shown in Fig. 4. Large filtering windows, on the other hand, collect the emission from all three peaks and reproduce the Rabi oscillations, which is the case selected for the Monte Carlo sampling. We explore in more details the opportunities offered by the Mollow triplet in Section 4.

### Effective quantum state

In this Section, we adapt the method of Zubizarreta Casalengua *et al*.^44^ to get the photon distribution *p*(*k*) of a frequency-resolved emitter, i.e., the probabilities for it to have *k* photons, which are, equivalently, the diagonal elements of its effective density matrix. Namely, the frequency-filtered source is regarded as an effective source of its own. Since filtering typically turns the original source into one of another kind, the effective source gets attributed a new annihilation operator *s* rather than the original *σ*. As we consider a photon source and we know that regardless of the original emitter, its filtering can produce an arbitrary number of photons, we assume that *s* is bosonic. The detector and the effective source are of course related. On the one hand, the normalized correlators are identical, since the detector measures faithfully the correlations of the source:

12 $${g}^{(n)}=\frac{\langle {s}^{\dagger n}{s}^{n}\rangle }{{\langle {s}^{\dagger }s\rangle }^{n}}=\frac{\langle {\xi }^{\dagger n}{\xi }^{n}\rangle }{{\langle {\xi }^{\dagger }\xi \rangle }^{n}},$$

and as, on the other hand, there is conservation of energy (assuming an ideal detector):

13 $${\gamma }_{\sigma }\langle {s}^{\dagger }s\rangle ={\rm{\Gamma }}\langle {\xi }^{\dagger }\xi \rangle \,,$$

since the rate of emission from the effective source is also that of detection. We find from combining Eqs (12) and (13) the relation between the unnormalized correlators:

14 $$\langle {s}^{\dagger n}{s}^{n}\rangle ={(\frac{{\rm{\Gamma }}}{{\gamma }_{\sigma }})}^{n}\langle {\xi }^{\dagger n}{\xi }^{n}\rangle \,,$$

**Figure 5:**
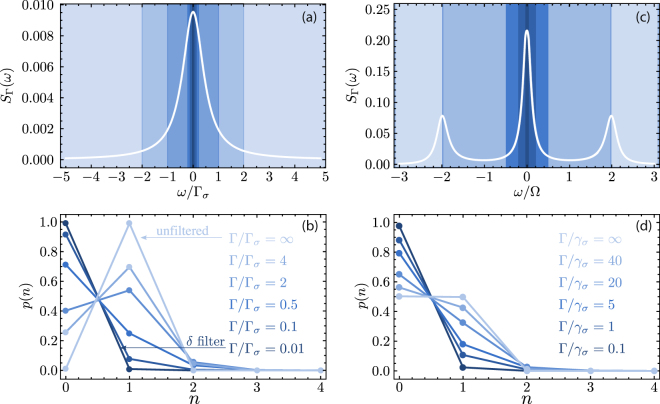
Effective quantum state reconstruction from the emission of a two-level system under high (**a**) incoherent and (**b**) coherent excitation. The various blue areas correspond to different filters linewidths. The light filtered in this way is equivalent to an unfiltered emitter whose quantum state has distribution *p*(*n*) to have *n* excitations, and is shown in (**b**) and (**d**), respectively. For the incoherently driven two-level system, case (a), the system is kept in its excited state by large pumping, so that without filtering, one observes a state close to the Fock state *p*(*n*) = *δ*_*n*,1_. Filtering leads to thermalization, with preponderance of vacuum but nonzero probability to detect $n > 1$ particles. The same is observed for the coherently driven two-level system, case (b), but starting from *p*(0) = *p*(1) = 1/2 due to the no-inversion of a two-level system in presence of stimulated emission. Parameters: for incoherent excitation, *P*_*σ*_ = 10^2^*γ*_*σ*_ (saturating the two-level system). For coherent excitation, Ω = 5*γ*_*σ*_. The rest of the parameters are as indicated in the figure.

The statistics *p*(*n*) of the photon emission from the effective source can now be obtained by inverting the relation $\langle {s}^{\dagger n}{s}^{n}\rangle ={\sum }_{k=n}^{N}(k!/(k-n)!)p(k)$ (with *N* large enough) to provide the probabilities *p*(*k*) for the effective source to have *k* quanta of excitation, for integer *k*. In the cascaded formalism, the correlators of the detectors, $\langle {\xi }^{\dagger n}{\xi }^{n}\rangle$ can be computed from the master equation, and are source dependent. The expressions for the population of a detector being fed by an incoherently and coherently driven two-level system are given by López Carreño and Laussy^32^ at resonance. Here, we provide a more general version for the detector at an arbitrary frequency. The incoherent case reads (note that there is a typo in Ref.^32^ with an extra factor 4; the correct result is as given here)

15 $$\langle {\xi }^{\dagger }\xi \rangle =\frac{{P}_{\sigma }({P}_{\sigma }+{\rm{\Gamma }}+{\gamma }_{\sigma })}{({P}_{\sigma }+{\gamma }_{\sigma })(({P}_{\sigma }+{\rm{\Gamma }}+{\gamma }_{\sigma }{)}^{2}+{{\rm{\Delta }}}^{2})}\,,$$

and the coherent excitation reads

$$\begin{array}{rcl}\langle {\xi }^{\dagger }\xi \rangle  & = & \mathrm{[2}{\gamma }_{01}{{\rm{\Omega }}}^{2}({\gamma }_{10}{({\gamma }_{11}^{2}+4{{\rm{\Delta }}}^{2})}^{2}({\gamma }_{12}^{2}+4{{\rm{\Delta }}}^{2})\\  &  & +\,4{{\rm{\Omega }}}^{2}({\gamma }_{10}{\gamma }_{11}^{2}{\gamma }_{12}{\gamma }_{32}+\mathrm{4(2}{\gamma }_{10}^{3}+16{\gamma }_{10}^{2}{\gamma }_{01}+23{\gamma }_{10}{\gamma }_{01}^{2}+8{\gamma }_{01}^{3}){{\rm{\Delta }}}^{2}\\  &  & -\,\mathrm{16(}{\gamma }_{10}-2{\gamma }_{01}){{\rm{\Delta }}}^{4})+32{\gamma }_{11}({\gamma }_{10}^{2}+4{{\rm{\Delta }}}^{2}){{\rm{\Omega }}}^{4})]\\  &  & /[({\gamma }_{10}^{2}+4{{\rm{\Delta }}}^{2})({\gamma }_{11}^{2}+4{{\rm{\Delta }}}^{2})({\gamma }_{01}^{2}+4{{\rm{\Omega }}}^{2})\\  &  & \times \,\{{\gamma }_{10}^{4}+6{\gamma }_{10}^{3}{\gamma }_{01}+12{\gamma }_{10}{\gamma }_{01}({\gamma }_{01}^{2}+2{{\rm{\Delta }}}^{2}+4{{\rm{\Omega }}}^{2})\\  &  & +\,{\gamma }_{10}^{2}\mathrm{[13}{\gamma }_{01}^{2}+\mathrm{8(}{{\rm{\Delta }}}^{2}+2{{\rm{\Omega }}}^{2})]\\  &  & +\,\mathrm{4[}{\gamma }_{01}^{4}+\mathrm{4(}{{\rm{\Delta }}}^{2}-2{{\rm{\Omega }}}^{2}{)}^{2}+{\gamma }_{01}^{2}\mathrm{(5}{{\rm{\Delta }}}^{2}+8{{\rm{\Omega }}}^{2})]\}]\,,\end{array}$$

with *γ*_*ij*_ ≡ *i*Γ + *jγ*_*σ*_. From a Monte Carlo simulation in a time *T*, the detector population is obtained as the ratio between the total number of clicks recorded and *γ*_*σ*_*T*. This allows to obtain the luminescence spectrum by scanning the detector in frequency (we do not show it but have checked it to be the case). One can now reconstruct the diagonal elements of the effective density matrix that, under an unspecified dynamics, is seen through the detector to yield the recorded photo-detection events. Since this can be achieved from (all) the Glauber correlators and the knowledge of the emitter’s mean population (known from the radiative lifetime), one can recover the effective *p*(*n*) in this way. This allows us to access new classes of quantum steady states, tailored by frequency-filtering. We now illustrate how this takes shape in the case already discussed of filtered two-level system emission, starting with the case of incoherent excitation. This is shown inFig. 5(a,b) for the filtered saturated two-level system, i.e., where the system is held in its excited state by very large pumping, ${P}_{\sigma }\gg {\gamma }_{\sigma }$, so that the density matrix reads *p*(0) → 0 and *p*(1) → 1. With Eq. (15) or from the detected clicks, one can compute the population and reconstruct this quantum state of the emitter, namely, the Fock state *p*(*n*) = *δ*_*n*,1_ (*δ* being the Kronecker function). The application of a filter turns the system from a two-level emitter to a source able to deliver more than one photon at a time, namely, for narrow enough filtering, *p*(*n*) ≈ (1 − *θ*)*θ*^*n*^ for all *n*, with *θ* ≈ 0.01 for the narrowest filter considered here. We have lost two orders of magnitude for the population but the probability to observe two (resp. three) particles is 1% (resp. 0.01%) of that to observe one only, which effectively shows how filtering a single-photon source turns it into a black body with nonzero probability to emit *n* photons. Its population is smaller than without the filter, as the latter is rejecting some photons, but the statistical distribution of those that go through now corresponds to an altogether different quantum state. A similar situation occurs with coherent excitation (when not filtering so much as to isolate the Rayleigh peak), with, for the case shown in Fig. 5(c,d)*, θ* ≈ 0.025. In both cases, one can see in this way at which point filtering prevents a single-photon source to emit non-classical states of light^45^, for instance by comparing *p*(1) to $3\sqrt{3}\mathrm{/(4}e)\approx \mathrm{47.8 \% }$, the smallest probability above which a state is non-Gaussian^46^.

## Tuneable statistics from the Mollow triplet

### Auto-correlations

**Figure 6:**
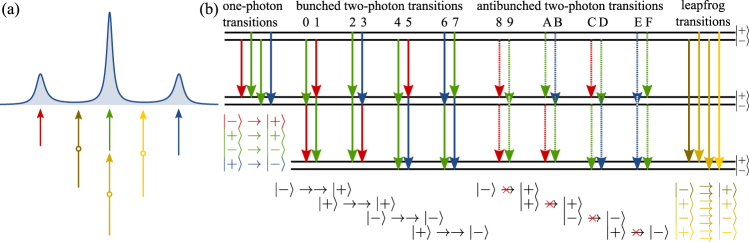
(**a**) Line shape and (**b**) level structure of the Mollow triplet. The lineshape is easily understood as the result of one-photon transitions between neighbour rungs of the dressed-state ladder, shown in red, green and blue. Since there are two degenerate green arrows, the central peak to which they correspond is twice as large. This picture is also helpful to visualise two-photon transitions. All the possible combinations are shown in (b). Combinations such as 8 and E, that occur only once, behave as expected. Other cases that occur multiple times, such as red and green transitions that happen in 0, 1, A and C, require exact computations to identify their actual behaviour. Also shown are the “leapfrog transitions”, that jump over the intermediate manifold in a direct two-photon emission. The cases where photons have the same frequency is shown in panel (a).

The Mollow regime that splits the luminescence into three lines provides in this way new natural spectral windows. One of the obvious questions this brings forward is: what is the statistics of the photons emitted by the three peaks? (is it the same as the total emission? One could imagine that all three peaks are emitting antibunched light since they ultimately originate from a two-level system. Our discussions so far in simpler systems should prepare us to find otherwise). The triplet structure, first computed exactly by Mollow^42^ but without providing a physical picture as to its origin, can actually be well understood from a simple model, introduced by Cohen-Tannoudji *et al*.: the “dressed atom” ^47^. In this model, the combined atom + laser is considered as a new entity, with a new structure of energy levels, shown in Fig. 6, and in which the transitions between the states account for the photoluminescence. On the basis of this picture, by considering two-photon transitions, one can foresee some correlations between the peaks. The transitions $|p\rangle \to |\pm \rangle \to |q\rangle$ for any *p*, *q* = + or −, that go down the Mollow ladder, could be expected to result in bunching. In contrast, since one cannot chain in this way $|p\rangle \to |-\rangle$ and $|+\rangle \to |q\rangle$ or $|p\rangle \to |+\rangle$ and $|-\rangle \to |q\rangle$, one can expect antibunching. Inspection of all the combinations of two-photon relaxations “suggests” that:

- each side peak is antibunched (cases 8 and E in Fig. 6),
- the central peak comes with both bunching (2 and 4) and antibunching (9 and F),
- the central peak comes with both bunching (0, 1, 6, 7) and antibunching (A, B, C, D) with each side peaks,
- the side peaks are bunched together (3, 5).

(One could also go further and consider time-ordering and/or detuning.) Early calculations by Apanasevich and Kilin^48^ and Cohen Tannoudji and Reynaud^47^ confirmed the side-peaks antibunching in autocorrelation and their mutual bunching in cross-correlations. In a later work with more involved calculations, Schrama *et al*.^49^ have shown that the central and side peaks feature no mutual correlations. This is due to interferences in the order of emission, that could nevertheless be linked to the co-existence of bunched and antibunched emission events. This shows that while intuition is strongly supported by the dressed-atom picture, it does not dispense from exact calculations for cases that could be ambiguous (and it confirms that there is indeed agreement with expectations for cases that cause no ambiguity, such as side-peaks emission).

With the theory of frequency-resolved photon correlations^11^, it is straightforward to compute such correlations exactly, like Mollow did for the luminescence spectrum, without referring to the dressed-states structure. This also allows to consider cases ouside their frequency windows, in fact, the complete landscape of two-photon correlations can be obtained^12,29^. In the case of the Mollow triplet^12,50^, it shows that the triplet structure reverberates at the two-photon level, through the apparition of a set of 3 hyperplanes, that obey the “leapfrog” equations

16 $${\omega }_{1}+{\omega }_{2}={\rm{\Delta }},\,{\rm{with}}\,{\rm{\Delta }}=-\,{{\rm{\Omega }}}_{+},0,{{\rm{\Omega }}}_{+}.$$

(The same applies at the *N*-photon level^50^). The triplet structure comes, at any photon-number level, from the three possibilities to join the two dressed initial and final states. Note that while there are four transitions, *N* is assumed sufficiently large for two transitions to be degenerate. The name of “leapfrog” comes from the fact that, at the *N*-photon level, transitions can occur by jumping over *N* − 1 intermediate manifolds. This relaxes energy conservation for the individual photons and restricts it to the combined emission. In this sense, this is a (*N* photons) version of Göpper Mayer’s two-photon processes^51^ that is however typically difficult to access (a notable exception is the planetary nebulae continuum^52^). The case *N* = 2 is shown on the right of Fig. 6. In the case of resonance fluorescence, such correlations have been noted by Apanasevich and Kilin^53^ for the $|\pm \rangle \rightrightarrows |\pm \rangle$ case (that is, overlooking the $|\pm \rangle \rightrightarrows |\mp \rangle$ counterparts, which are equally obvious with the dressed atom picture in mind). Interestingly, part of this school of researchers, who has produced noteworthy works on the problem of photon correlations^54–56^, has recently expressed some critics on this leapfrog picture, writing that^28^ “*the concept of the “leapfrog” processes is not justified*”, that they “*present an alternative explanation*” based on “*the unnormalized spectral correlation function*” which is, they write, “*a true measure of spectral correlations*” and “*which exhibits no signatures of the leapfrog transitions*”. From their discussion, one thus understands that the production of strongly-correlated photons away from the peaks, that we predict, is an artifact due to normalization. In principle, one can indeed inflate the vacuum and create what one could regard as an artificial superbunching. This is not what happens with leapfrog emission, however, although according to these authors, nothing of interest takes place away from the peaks, what they illustrate by producing a two-photon spectrum remarkably featureless, in contrast to our two-photon spectrum that is rich from photon correlations flourishing away from the peaks. Their non-normalized spectrum is correct but, we believe, is also not interesting as it merely shows that first order processes smother second-order ones, as is however well-known and expected. We show, in contrast, that the scarce signal from higher-order processes has stronger correlations than those from first-order processes. This will be amply and vividly illustrated through Monte Carlo simulations below. One can also consider placing a cavity in this “featureless” region when not normalized, and observe how the system then keeps emitting strongly correlated photons but now dominating over the other first-order processes^10,57^, which would not happen would the correlation be an artifact due to normalization. We will show below thanks to the frequency-resolved Monte Carlo simulations how one can anyway see the manifestation of leapfrog processes with the naked eye. The rest of their discussion is only semantics, in which case we should clarify, as this is apparently needed, that the dressed-atom picture is, precisely, a “picture”, that is, an insightful mental representation that is helpful to visualize the basic mechanism at play, support the intuition and guide further inquiries. This does not preclude exact calculations based on the opaque equations of quantum mechanics. This is possibly why the Mollow triplet is named after Benjamin, not only for his seminal input but also in recognition of the exact expression, although the Cohen Tannoudji and Reynaud approximate picture is the one everybody has in mind when thinking about this problem. We combine both approaches: the sensor technique provides the exact result, while the leapfrog processes provide a physical representation *à la* Cohen Tannoudji *et al*. Thus, in the same way that resonance fluorescence is not spontaneous emission from the dressed atom, the leapfrog emission is not, strictly speaking, spontaneous emission jumping over intermediate manifolds. This is, instead, a complicated process that involves the laser and the two-level system in a sequence of coherent absorption and emission. We have in fact shown^33^ how even fluorescence in the low-driving regime does not consist of Rayleigh scattering events but form an intricate interference between emission and absorption, that powers the single-photon emission mechanism by suppressing the fat tails of the Lorentzian and turning the lineshape into a *t*_2_ distribution instead. In the strongly nonlinear regime, a similar dynamics takes place but it becomes forbidding and certainly not even useful to apprehend the problem in these terms. Note that although inspired by the dressed-atom approach of the problem, ultimately, our computations are exact (and in full agreement with this physical picture). While the dressed atom has proven to be extremely fruitful for their purpose, we find it to be even more so in our *N*-dimensional case^50^, if not mandatory. It is, indeed, very easy based on this concept to understand why some configurations have less strong correlations than others, for instance, one of the $|+\rangle$ ⇶ $|+\rangle$ transitions with one photon non-degenerate with the two others (of interest for photon-heralding of two-photon emission), is particularly weak. This is because it is resonant with the $|-\rangle \to |+\rangle \rightrightarrows |-\rangle$ transition, that breaks this channel of relaxation by interposing a real state in the three-photon emission. Configurations $|+\rangle$ ⇶ $|+\rangle$ whose energies do not intersect with real states, on the other hand, have very strong correlations and are more suitable for heralding purposes. It would seem difficult to make sense of these observable facts, that follow from exact computations, without the leapfrogging concept. In fact we can easily generalise them to arbitrary photon orders and guess which energies are to be avoided in a *N* dimensional space to harness the best sought configuration of multi-photon emission, without having to undertake any actual computation. Is the concept therefore not justified and should one only be allowed infinite series of Feynman diagrams? We believe that the comments of Shatokhin *et al*. targetting the leapfrog picture bring very little to the discussion, if not in fact muddying it with confused statements and blurring their actual technical contributions that otherwise concur with our results, and of which we wish to remove nothing, as this approach has its merits.

**Figure 7:**
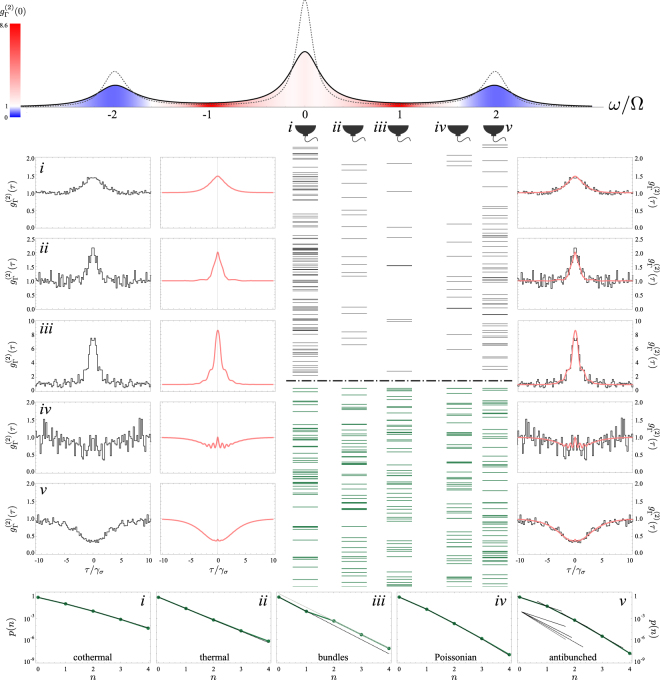
Frequency-resolved Monte Carlo simulation of the Mollow triplet in independent frequency windows. The dotted (solid) lineshape is the triplet as detected by an ideal (finite-bandwidth Γ) detector. Sequences of clicks in the frequency windows *i*–*v* have been recorded, with 17241, 22836, 99457, 9112 and 46126 events, respectively. Small samples are displayed, in the same time window (black ticks, up) or with renormalization of time to compare equal intensities (green ticks, bottom). Clear structures are visible even to the naked eye, in particular, the existence of leapfrog emission is obvious. Only one detector has been used, so the streams are not here cross-correlated. The autocorrelations are shown as measured by the Monte Carlo data (left column), as computed by the theory of frequency-resolved photon correlation (2nd column, red) and both superimposed (right column), to show their rich fine structure and the overall agreement. The effective quantum state reconstruction is shown at the bottom, together with fits to fundamental distributions. Panel *v* has its successive emission probabilities brought together to show the exponential extinction of higher photon-numbers. 1/*γ*_*σ*_ sets the unit, Ω = 5*γ*_*σ*_ and Γ = *γ*_*σ*_. The clicks correspond to the emission events of the operators *c*_4_ and *c*_5_ in Eq. (18b).

Back to the general discussion. We show in Fig. 7 the statistics of clicks from photo-detection events of the Mollow triplet in frequency windows spanning from the central peak to the side peaks, including various other windows in between, in particular, the leapfrog window. Note that, here as well, the data is a single-detector observable, that is to say, the different streams shown are not correlated to each others as they have been obtained by the same detector in different runs of the experiment. It would require 5 detectors to obtain the same result but with correct cross-correlations (this is beyond the scope of the present discussion that will go up to two detectors only, but is of course a topic of interest for applications). As we did in Fig. 3, we show both ticks in a given time window (in black) and with a rescaling of the unit of time so that their densities are equal (in green). Here as well, the relative emission rates mean that longer integration times are required when collecting away from the peaks. The gain in terms of correlation strengths, however, makes it worthwhile to focus on these regions of reduced emission, in a spirit akin to distillation: trading quantity for quality^29^. The frequency windows have been chosen as they correspond to particular cases of interest:

i. Photons from the central peak.
ii. Case where ${g}_{{\rm{\Gamma }}}^{\mathrm{(2)}}\mathrm{(0)}=2$ (usually attributed to thermal light).
iii.  Photons from leapfrog emission.
iv. Case where ${g}_{{\rm{\Gamma }}}^{\mathrm{(2)}}\mathrm{(0)}=1$ (usually attributed to coherent or uncorrelated light).
v. Photons from a side peak.

The central peak is partially thermalized, with a *g*^(2)^(*τ*) that closely resembles the form of thermal fluctuations, *g*^(2)^(*τ*) = 1 + exp(−2|*τ*|/*τ*_0_). Upon closer inspection, however, this is an approximation as the exact solution presents small departures, in particular, a differentiable slope at the origin and small ripples that are thinly visible on the theory curve, that we keep separate from the Monte Carlo data for clarity (the quality of their agreement is shown in the rightmost column). Note that the dynamics of coherent driving of a two-level system is considerably more complicated than its incoherent counterpart and we could not find, so far, a general closed-form expression for ${g}_{{\rm{\Gamma }}}^{\mathrm{(2)}}(\tau )$ in this case for arbitrary frequencies. Applying the technique of effective-quantum state reconstruction from the correlators, described in Section 3.3, we find that the statistics *p*(*n*) fits well with a cothermal distribution with ≈80% of thermal emission and ≈20% of uncorrelated emission. Overall, the emission of the central peak is thus well described by a mixture of thermal and uncorrelated light. It is, as such, not very interesting per se. Turning now to the second frequency window, *ii*, which features ${g}_{{\rm{\Gamma }}}^{\mathrm{(2)}}\mathrm{(0)}=2$, characteristic of thermal emission, one can now see more clearly the deviation from the thermal paradigm, with bulging and tails deforming the correlation function. These are well reproduced by the Monte Carlo statistics and we let the reader decide if their statistical acuity lets them, on the small sample of clicks reported here, observe deviations from the thermal paradigm (cf. Fig. 3*viii*).

The most interesting window, *iii*, lies halfway between the central and side peak. This is the frequency at which, according to our interpretation of the theory^12^, two photons can make a leapfrog process from the state $|+\rangle$ in a given manifold to the state $|-\rangle$ two manifolds below, jumping over the intermediate manifold, cf. the rightmost transition in Fig. 6. These photons are strongly correlated in several ways. From a photo-detection point of view, they should arise as more occurrences of closely-spaced two-photon clicks than if the emission was uncorrelated. In particular, their rate of coincidences should increase, leading to ${g}_{{\rm{\Gamma }}}^{\mathrm{(2)}}\mathrm{(0)}\gg 2$, or so-called superbunching. This is both predicted by the exact theory^11,12^ and observed in our Monte Carlo simulations, as seen in Fig. 7. Remarkably, even with as few as 9112 clicks collected in the numerical experiment, we can reconstruct a high-quality signal, revealing the fine details of its structure. Note as well that on the real-time series of clicks, out of the nine photons emitted, four came as two-photon bundles (the fifth and sixth clicks are so closely spaced as almost overlapping; other ticks are single-photon events). The small sample of clicks also shows strong ordering, combining equal spacing and gaps of no emission. While the latter is characteristic of thermal emission, the former is typically characteristic of antibunching. This combination can be seen as the selection through filtering of strongly correlated emission from the emitter, rather than tampering from the filters on the statistics: focusing to these frequency windows allows us to detect the two-photon emission events that occurs, from the dressed-atom picture, at this frequency. It would be rewarding to apply this technique to the filtered emission of a “bundler”^10^, a device that emits the majority, and in some regime, close to 100%, of its light as *N*-photon emission, and for which filtering has been shown to considerably boost the purity of the quantum emission^57,58^. Also further photon-counting characterization would certainly be enlightening, and preliminary investigations show that the percentage of closely-spaced photons is over an order of magnitude higher in *iii* than in the others at the exception of *ii*, as compared to which it is only about 3.8 times larger. We leave further characterizations for future works, but provide a last compelling manifestation of leapfrog emission from the effective quantum state reconstruction approach, cf. Section 3.3. This highlights the frequency window *iii* as the most dissimilar one as compared to the others, featuring a neat kink at the probability to have two photons, *p*(2), showing the relative predominance of two-photon emission. Overall, this simulation makes it obvious that the emission in this frequency window suffers from no artifact of post-selection or normalization, but does indeed provide strongly correlated photon streams.

The fourth frequency window, *iv*, chosen for its ${g}_{{\rm{\Gamma }}}^{\mathrm{(2)}}\mathrm{(0)}=1$ of uncorrelated emission, is also a case that shows strong departures at nonzero *τ* due to filtering. This is, here again, well captured by the Monte Carlo clicks and is visibly noticeable on the small sample, that features ordered clumps of uncorrelated clicks. With the last window, *v*, we come back to a case well studied in the literature, of antibunched emission, albeit far from perfect (${g}_{{\rm{\Gamma }}}^{\mathrm{(2)}}\mathrm{(0)}\approx 0.42$ and ${min}_{\tau }\,{{\rm{g}}}_{{\rm{\Gamma }}}^{(2)}(\tau )\approx 0.37$). The fact that the minimum antibunching is not at zero is another manifestation of frequency filtering, thinly visible on the figure as small oscillations, but not reproduced at this level of signal by the Monte Carlo data. Correspondingly, the *p*(*n*) shows increasingly suppressed probabilities to get higher number of photons.

### Cross-correlations

In this final part, we consider cross-correlations, for which the Mollow triplet is also a particularly suitable lineshape. That is to say, we consider two detectors acquiring data simultaneously. The master equation for two detectors upgrade Eq. (5) to:

17 $${{\rm{\partial }}}_{t}\rho =i[\rho ,{H}_{\sigma }+\sum _{i=1,2}{\omega }_{{\xi }_{i}}{\xi }_{i}^{\dagger }{\xi }_{i}]+\frac{{\gamma }_{\sigma }}{2}{{\mathscr{L}}}_{\sigma }\rho +\sum _{i=1,2}(\frac{{{\rm{\Gamma }}}_{i}}{2}{{\mathscr{L}}}_{{\xi }_{i}}\rho +\sqrt{{\alpha }_{i}{\gamma }_{\sigma }{{\rm{\Gamma }}}_{i}}\{[\sigma \rho ,{\xi }_{i}^{\dagger }]+[{\xi }_{i},\rho {\sigma }^{\dagger }]\})\,,$$

with *ξ*_1_, *ξ*_2_ the two detectors. The factors *α*_1_ = (1 − *χ*_0_ − *χ*_1_)(1 − *χ*_2_) and *α*_2_ = *χ*_0_(1 − *χ*_3_), satisfying simultaneously 0 ≤ *χ*_0_, *χ*_1_, *χ*_2_, *χ*_3_ ≤ 1 and *χ*_0_ + *χ*_1_ ≤ 1, take into account the several decay channels of the source: a fraction *χ*_1_ into free space, a fraction *χ*_0_ to the detector *ξ*_1_ and the remaining fraction (1 − *χ*_0_ − *χ*_1_) to the detector *ξ*_2_. In analogy with the case of a single detector, the system described by the master equation (17) has five collapse operators:

18a $${c}_{1}=\sqrt{{\chi }_{0}{\gamma }_{\sigma }}\,\sigma +\sqrt{(1-{\chi }_{2}){{\rm{\Gamma }}}_{1}}{\xi }_{1}\,,\,{c}_{2}=\sqrt{(1-{\chi }_{0}-{\chi }_{1}){\gamma }_{\sigma }}\,\sigma +\sqrt{(1-{\chi }_{3}){{\rm{\Gamma }}}_{2}}{\xi }_{2}\,,\,{c}_{3}=\sqrt{{\chi }_{1}{\gamma }_{\sigma }}\,\sigma ,$$

18b $${c}_{4}=\sqrt{{\chi }_{2}{{\rm{\Gamma }}}_{1}}{\xi }_{1}\,\,{\rm{a}}{\rm{n}}{\rm{d}}\,\,{c}_{5}=\sqrt{{\chi }_{3}{{\rm{\Gamma }}}_{2}}\,{\xi }_{2},$$

and its associated non-hermitian Hamiltonian becomes

19 $$\mathop{H}\limits^{ \sim }={H}_{\sigma }+{H}_{{\xi }_{1}}+{H}_{{\xi }_{2}}-i(\sqrt{{\alpha }_{1}{\gamma }_{\sigma }{{\rm{\Gamma }}}_{1}}\,{\xi }_{1}^{\dagger }\sigma +\sqrt{{\alpha }_{2}{\gamma }_{\sigma }{{\rm{\Gamma }}}_{2}}\,{\xi }_{2}^{\dagger }\sigma )-\frac{i}{2}({\gamma }_{\sigma }{\sigma }^{\dagger }\sigma +{{\rm{\Gamma }}}_{1}{\xi }_{1}^{\dagger }{\xi }_{1}+{{\rm{\Gamma }}}_{2}{\xi }_{2}^{\dagger }{\xi }_{2}).$$

**Figure 8:**
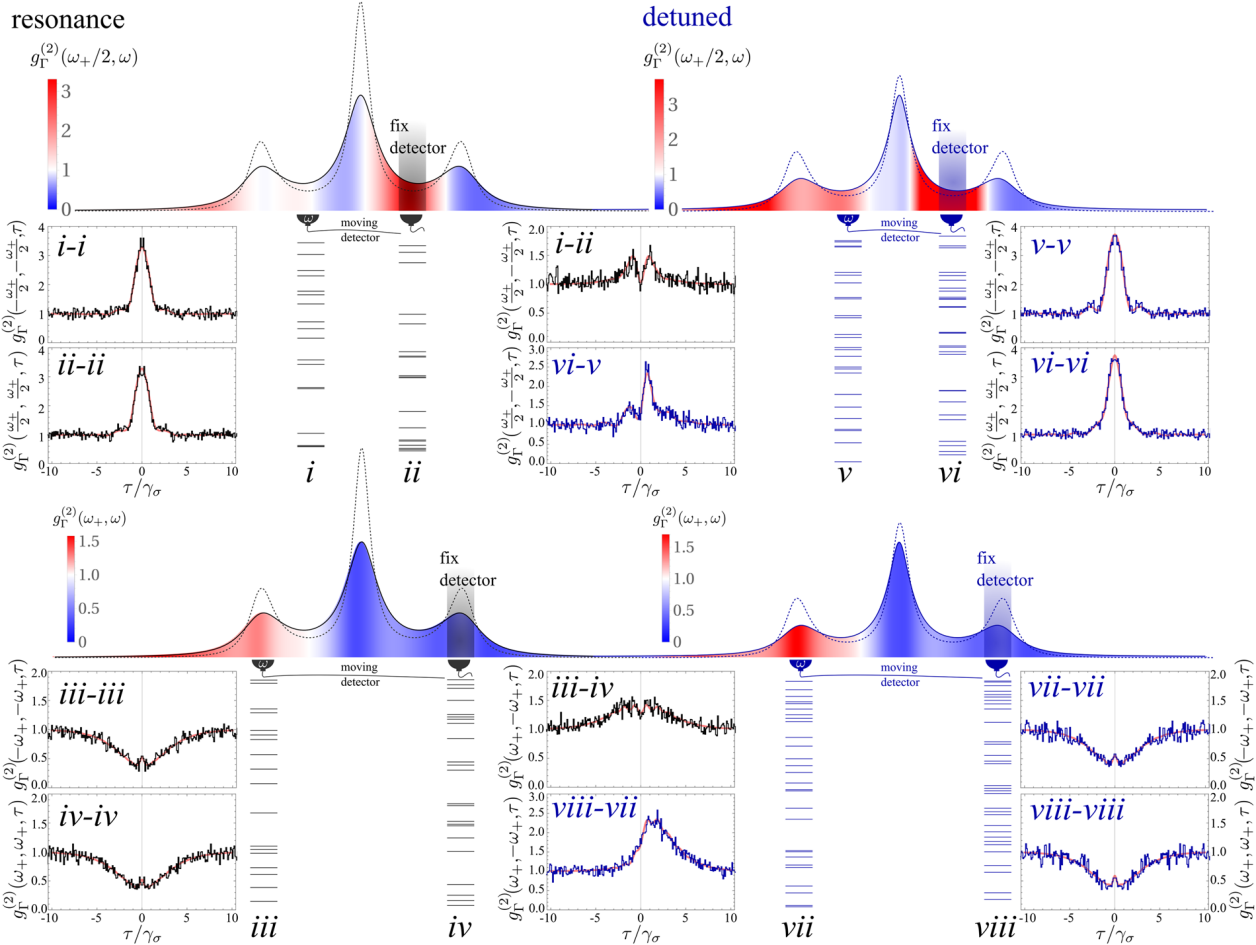
Frequency-resolved Monte Carlo simulation of the Mollow triplet in dual frequency windows. The case on the left (right) is for driving the two-level system at resonance (with detuning Δ = 1.5*γ*_*σ*_). The dotted (solid) lineshape is the triplet as detected by an ideal (finite-bandwidth Γ) detector. The color code within the spectra is for one filter kept fixed at a leapfrog window (top case) or at a sideband (bottom case). Sequences of clicks have been recorded in the windows *i* and *ii* and *iii* and *iv*, the two groups being independent (that is, clicks between, e.g, *i* and *iii* are not correlated). Small samples are displayed, with renormalization of time to compare equal intensities between the two groups (time is the same within each group). Strong correlations of photons with different frequencies are clear, in particular, the simultaneity of leapfrog emission and their heralding character with detuning are obvious. The cross-correlations are shown as measured by the Monte Carlo data (left column) and as computed by the theory of frequency-resolved photon correlation (2nd column, red). 1/*γ*_*σ*_ sets the unit, Ω = 5*γ*_*σ*_, Δ = 1.5*γ*_*σ*_ and Γ = *γ*_*σ*_. The clicks correspond to the emission events of the operators *c*_4_ and *c*_5_ in Eq. (18b).

As for the case of autocorrelations, one could similarly demonstrate the equivalence between cross-correlations to any orders as computed through the frequency-resolved photon correlations and those obtained through Eq. (17) above. Also as was done before for single frequency windows, by applying the Monte Carlo techniques to the detectors, one can thus obtain simulated photon emissions, this time in two frequency windows. Computing the correlations from this raw data provides a numerical version of the theoretical correlations. This is shown in Fig. 8 for the joint emission of the two sidebands on the one hand, and then of the two leapfrog windows on the other hand, both when driving the two-level system at resonance or with a detuning.

While we considered a small subset only of the possible autocorrelations in Fig. 7 for the Monte Carlo data, we could still provide a comprehensive theoretical result at least for ${g}_{{\rm{\Gamma }}}^{\mathrm{(2}}\mathrm{(0)}$ through the color-coded spectrum. For cross-correlations, however, this would require a 2D plot to reproduce the entire two-photon correlation spectrum^12^. Instead, we consider here the case where one detector is fixed and the other one sweeps the rest of the spectrum, providing the cross-correlations. We then place the other detector for Monte Carlo sampling at the location of interest. As before, we show raw photon emission, but with no time-rescaling as the respective frequencies chosen have similar intensities. We also compare two-photon correlations computed from this data (black and blue lines, at resonance and with detuning respectively) with the theoretical result (red lines). The main difference between these cases and the previous ones is that the two streams of photons are now correlated as the detectors are measuring simultaneously. If restricting to one stream only, we recover the previous cases, so in Fig. 8, panels *i*-*i* and *ii*-*ii* on the one hand, as well as *iii*-*iii* and *iv*-*iv* on the other hand, can be found in Fig. 7 (in panels *iii* and *v* respectively). The two cases have different parameters, since with two detectors, the simulation is more computer intensive and we chose a case that provides more emission between the peaks. One can check however how the qualitative shapes of the correlations remain the same. At resonance, both streams provide the same type of correlations, but with detuning they could be different. This is indeed the case for the leapfrog emission, and small departures can be observed between *v*-*v* and *vi*-*vi*, both in the theoretical line and the Monte-Carlo generated data: the oscillations are more marked for the low-energy window and a depletion is indeed visible in *vi*-*vi* that disappeared in *v*-*v*. The side peaks, however, feature similar correlations. This shows again the typical richer dynamics away from the peaks.

Both at resonance or with detuning, what is of interest when detecting in different windows simultaneously is their cross-correlations, as shown in the central column of Fig. 8 with panels *i*-*ii*, *vi*-*v*, *iii*-*iv* and *viii*-*vii*. There are now clear features in these cross-correlations, whereas the same procedure applied to the streams of the previous cases features no correlations, i.e., one obtains flat lines. At resonance, the cross-correlations are symmetrical in time. The side peaks correlations feature tiny oscillations which are however too small to be observed with the amount of signal we acquired in the numerical experiment, and they are hidden by its fluctuations. With detuning, they can and do become time-asymmetrical, as shown in panels *vi*-*v* and *viii*-*vii*. In such cases, the order of detection matters, and in both cases, the detection on first detector, *vi* or *viii*, respectively, makes it more likely to later detect (with around 1/*γ*_*σ*_ delay) a photon on the second detector, *v* and *vii*, respectively. The strength of such correlations, less than 3, is still fairly modest to call this heralding, but this is the basis for such a mechanism to be exploited with proper engineering, such as Purcell-enhancement. Like before, our procedure yields correlated streams of photons of different frequencies, that we have just shown through their agreement with the theory of frequency-resolved photon correlations, simulate the actual photon emission from the system. One could use this raw data to compute numerically, e.g., counting or time-delay distributions, otherwise not easily accessible theoretically. Of course, the scheme could in principle be extended to any number of detectors and allow consequently higher orders of correlation to be computed in this way. In the limit of an infinite number of detectors, each with a given frequency and vanishing spectral width, one would thus simulate the ideal emission of the system. With a finite number of detectors with a finite bandwidth, one would simulate its filtered emission. We believe that a complexity analysis of the correlations would allow to use the emission of the two-level system as a simpler platform than boson sampling^59^ to test quantum supremacy by making a laboratory measurement which no classical computer would be able to simulate.

## Conclusions and Perspectives

In conclusion, we have presented computer experiments that simulate numerically the photon emission from a quantum emitter, specifically, a two-level system under both coherent and incoherent driving, at both low and large pumping. Our approach is based on the Quantum Monte Carlo technique^5–8^ applied to the cascaded formalism^36,37^. We have shown how the correlations computed from the raw data of the simulation match with the theoretical results provided by the theory of frequency-resolved photon correlations^11^. In the simplest case, we have shown how filtering spoils antibunching and turns a two-level system into a thermal source, albeit in a more subtle way than is usually assumed. We have also shown more generally how frequency filtering provides a resource to tailor and engineer photon statistics, in particular thanks to its selection of strongly correlated processes such as “leapfrog” transitions that consist in the simultaneous emission of a photon bundle between two non-contiguous dressed states in the level structure of the system^12^. This displays rich and potentially useful features that are captured in the Monte Carlo simulation and that would be similarly observed experimentally. An apparent shortcoming is that the signal is scarce in frequency windows that are the most strongly correlated. This is however a direct consequence of dealing with the quantum part of the signal: there is less of it. Frequency filtering acts as a process akin to distillation, with the same consequence of providing quality at the expense of quantity^29^. Nevertheless, quantum engineering can come at the rescue and already the oldest trick of cavity QED–Purcell enhancement–allows, in some regime, to have *all* the light of the system emitted as strongly correlated photons^10^. Using cavities to Purcell-enhance leapfrog processes, one can devise new generations of heralded *N*-photon sources, or, even more generally, bring the system to emit in any desired distribution of photons^50^. Such configurations remain to be studied in detail and, of course, implemented in the laboratory. This should provide one route for universal multi-photon sources, with heralded *N*-photon sources as the most elementary realization. Since leapfrog processes are energy-conserving *N*-photon relaxations, they also appear particularly suitable for energy-time entanglement emission, that power a class of quantum-cryptographic protocols with technical advantages as compared to those based on entangling in polarization. The latest work from Peiris *et al*.^60^, who is so far leading the laboratory implementation of this emerging branch of quantum optics, focused on the side peaks emission and, as a consequence, failed to break the barrier of a Bell violation. This has been argued no to be a proof of nonlocality anyway^61^ due to its 50% post-selection^62^. It is easily computed that leapfrog emission would break the Franson limit, but in the light of the Franson configuration’s loophole^63^, the new challenge is to turn to stricter conditions of Bell violations such as Chained Bell’s inequalities. While this has been recently demonstrated^64^, the tunable statistics from the Mollow triplet and its windows of strong correlations make it a promising platform to further test and advance this line of research. Finally, the combinatoric aspects that quickly make such simple problems numerically forbidding also suggest that a two-level system could be used in the laboratory for tests of quantum supremacy directly from photon detections, without a complex system of beam splitters intervening to bring in the quantum complexity^65,66^. All these results leave much for room for further works, and we foresee that frequency-resolved photon correlations will become a major theme of photonics. They are relevant even when they are ignored and awareness of the underlying physics should allow to considerably optimize, tune and expand the range of applications of quantum light sources.
